# Activity of Synthetic Peptide KP and Its Derivatives against Biofilm-Producing *Escherichia coli* Strains Resistant to Cephalosporins

**DOI:** 10.3390/antibiotics13080683

**Published:** 2024-07-24

**Authors:** Lorenza Artesani, Tecla Ciociola, Alice Vismarra, Cristina Bacci, Stefania Conti, Laura Giovati

**Affiliations:** 1 Department of Medicine and Surgery, University of Parma, 43126 Parma, Italy; lorenza.artesani@unipr.it (L.A.); tecla.ciociola@unipr.it (T.C.); stefania.conti@unipr.it (S.C.); 2 Microbiome Research Hub, University of Parma, 43124 Parma, Italy; 3 Department of Veterinary Science, University of Parma, 43126 Parma, Italy; alice.vismarra@unipr.it (A.V.); cristina.bacci@unipr.it (C.B.)

**Keywords:** antimicrobial peptides, *Escherichia coli*, biofilm, extended-spectrum β-lactamases, confocal laser scanning microscopy

## Abstract

Bacterial resistance to β-lactam antibiotics, particularly new generation cephalosporins, is a major public health concern. In *Escherichia coli*, resistance to these antibiotics is mainly mediated by extended-spectrum β-lactamases (ESBL), which complicates a range of health-threatening infections. These infections may also be biofilm-related, making them more difficult to treat because of the higher tolerance to conventional antibiotics and the host immune response. In this study, we tested as potential new drug candidates against biofilm-forming ESBL-producing *E. coli* four antimicrobial peptides previously shown to have antifungal properties. The peptides proved to be active in vitro at micromolar concentrations against both sensitive and ESBL-producing *E. coli* strains, effectively killing planktonic cells and inhibiting biofilm formation. Quantitative fluorescence intensity analysis of three-dimensional reconstructed confocal laser scanning microscopy (CLSM) images of mature biofilm treated with the most active peptide showed significant eradication and a reduction in viable bacteria, while scanning electron microscopy (SEM) revealed gross morphological alterations in treated bacteria. The screening of the investigated peptides for antibacterial and antibiofilm activity led to the selection of a leading candidate to be further studied for developing new antimicrobial drugs as an alternative treatment against microbial infections, primarily associated with biofilms.

## 1. Introduction

Microbial infections pose a major threat to public health in relation to the emergence and spread of antimicrobial resistance [1]. This phenomenon is driven by the natural adaptation of microorganisms to ecological pressure but is accelerated by the inappropriate use of antimicrobial drugs [2]. *Escherichia coli*, a Gram-negative bacterium belonging to the *Enterobacteriaceae* family, has a high potential for spreading among animals, humans, and the environment [3]. The species includes both recognized pathogens and members of the human and animal intestinal microbiota, as well as bacteria capable of colonizing the environment and contaminating food [2]. These microorganisms also serve as a significant reservoir for resistance genes, carried by mobile genetic elements like plasmids and transposons, which can be transferred horizontally between different *E. coli* strains and other bacterial species [4].

The ability of drug-resistant *E. coli* to infect and colonize various hosts is particularly concerning due to the spread of genes that encode inactivating enzymes, such as extended-spectrum β-lactamases (ESBL), which can hydrolyze cephalosporins [5,6,7]. ESBL-producing *E. coli* can also form biofilms, which are organized communities of microbial cells adhered to a surface and surrounded by a self-produced matrix of extracellular polymers that protects them from the immune system and antimicrobial compounds [7,8,9,10].

To overcome antimicrobial resistance, adjunctive and/or alternative therapeutic strategies against infectious diseases are urgently needed. Among the different approaches, natural or synthetic antimicrobial peptides (AMPs) of various origins arouse great interest as possible anti-infective drugs due to their mechanisms of action, which are generally less susceptible to the widespread development of resistance [11].

Natural AMPs are found in a wide range of organisms, act as a primary defense against pathogens, and many of them also display immunoregulatory properties that are essential for antimicrobial efficacy in vivo [12,13,14]. Some AMPs can prevent biofilm formation and/or eradicate preformed biofilms [15,16,17,18]. The mechanisms associated with their antibiofilm activity include reducing attachment by inhibiting swimming/swarming motility or interfering with flagellar assembly, perturbing the cell wall or membrane, promoting biofilm dispersal by stimulating twitching motility, and altering gene regulation or quorum sensing [15,16,17,18]. Importantly, many AMPs are active against biofilms of multi-drug resistant microorganisms [19,20,21].

In recent years, advancements in synthesis and delivery technologies have facilitated the development of AMPs, with some of them now in clinical trials [22]. Additionally, research has focused on modifying AMP sequences to enhance their antimicrobial potency, stability, or selectivity [23]. In this context, the sequence of the broad-spectrum killer peptide KP [24] was used to rationally design two groups of synthetic derivatives. Among these, K10S, obtained by replacing the first KP residue (alanine) with lysine [25], and the in silico predicted derivatives K10T-TT and K10S-SS [26] showed promising activity as antifungals and were well characterized for their structure-activity relationship [25,26].

The aim of this study was to screen these peptides against both planktonic cells and biofilms of ESBL-producing *E. coli* isolates to identify a leading candidate for the development of alternative treatments for biofilm-associated infections caused by resistant bacteria. The activity of KP, K10S, K10T-TT, and K10S-SS was assessed against the cephalosporin-sensitive *E. coli* ATCC 25922 reference strain and the selected cephalosporin-resistant *E. coli* isolates obtained from animals in intensive farming environments [27].

## 2. Results

### 2.1. Bactericidal Activity against Planktonic E. coli Cells

The bactericidal activity of the investigated peptides (Table 1) against the cephalosporin-sensitive *E. coli* ATCC 25922 reference strain and the selected ESBL-producing *E. coli* isolates was evaluated in vitro by a colony-forming unit (CFU) assay. As shown in Table 2, all the investigated peptides were effective against both the cephalosporin-sensitive reference strain and the ESBL-producing isolates, with obtained half-maximal effective concentrations (EC_50_) ranging from 0.051 to 1.099 × 10^−6^ M.

### 2.2. Inhibition of Biofilm Formation on Polystyrene Surfaces

The ability of the peptides to interfere with early stages of biofilm formation by the tested *E. coli* strains on polystyrene plates was investigated using the crystal violet (CV) assay. KP and K10S, although active against planktonic cells, did not show a significant inhibition of biofilm formation. KP showed the highest activity against *E. coli* FS3, inhibiting biofilm formation of 36% and 15% at 1.0 × 10^−4^ M and 0.5 × 10^−4^ M, respectively. K10S showed the highest inhibition of biofilm formation by *E. coli* ATCC 25922, with an EC_50_ value of 43.477 × 10^−6^ M, although with very low confidence (confidence interval 1.530–1235.7). In contrast, treatment with K10T-TT and K10S-SS caused a significant dose-dependent reduction in biofilm biomass, with EC_50_ values in the order of 10^−5^ M (Table 3).

### 2.3. Activity of K10T-TT on Mature E. coli Biofilms Formed on Stainless Steel Surfaces Assessed by Confocal Microscopy 

**Figure 1:**
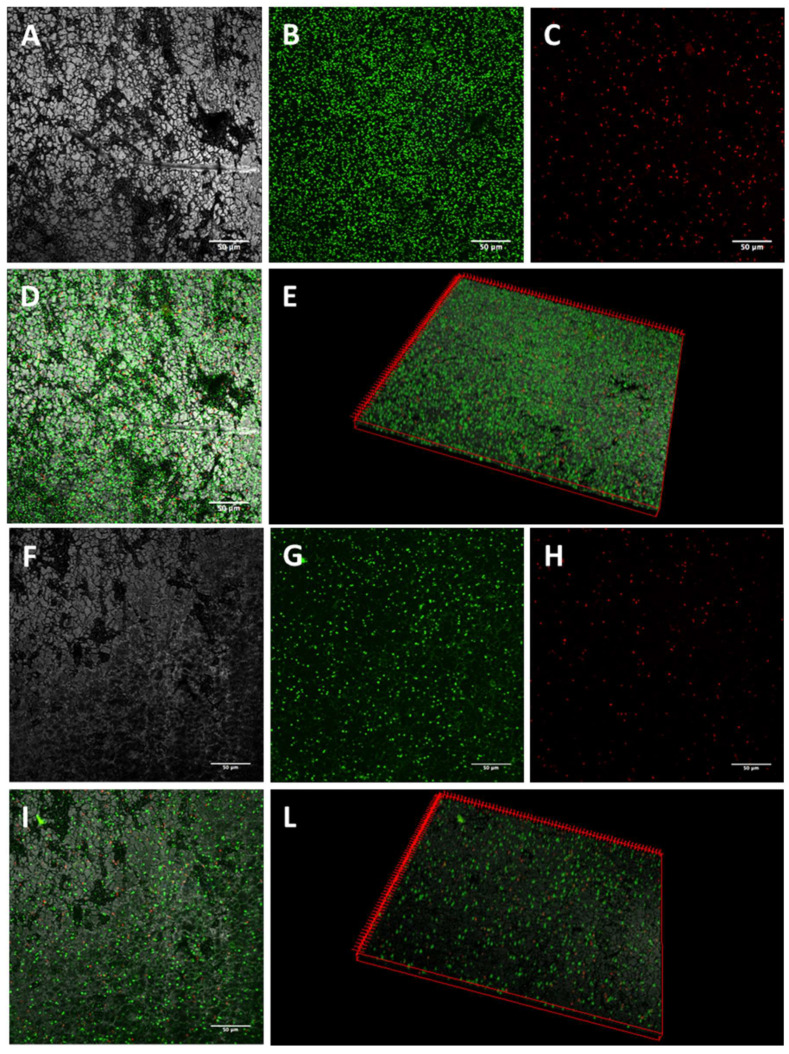
Representative images of *E. coli* ATCC 25922 biofilms obtained by CLSM. Mature biofilms formed on stainless steel surfaces were treated for 16 h with 46 µM K10T-TT (**F**–**L**) or sterile water (**A**–**E**). (**A**,**F**): stainless steel surface (reflected light); (**B**,**G**): viable cells (green fluorescence); (**C**,**H**): dead cells (red fluorescence); (**D**,**I**): merge of green and red channels; (**E**,**L**): three-dimensional reconstructions of the series along the Z-axis. Bars, 50 µm.

**Figure 2:**
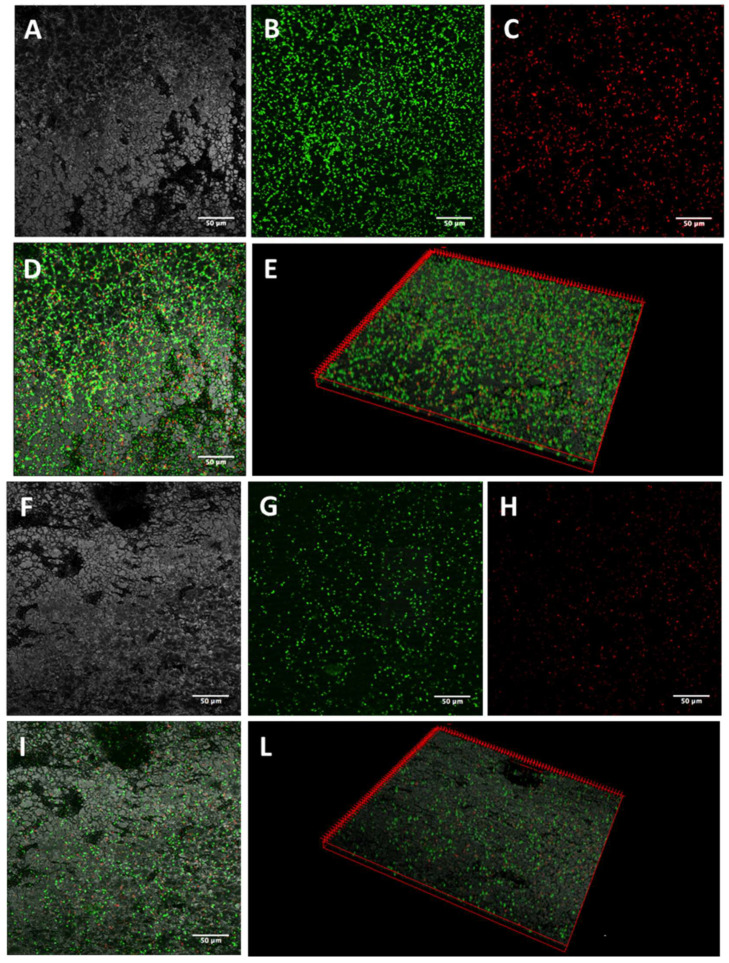
Representative images of *E. coli* FS3 biofilms obtained by CLSM. Mature biofilms formed on stainless steel surfaces were treated for 16 h with 46 µM K10T-TT (**F**–**L**) or sterile water (**A**–**E**). (**A**,**F**): stainless steel surface (reflected light); (**B**,**G**): viable cells (green fluorescence); (**C**,**H**): dead cells (red fluorescence); (**D**,**I**): merge of green and red channels; (**E**,**L**): three-dimensional reconstructions of the series along the Z-axis. Bars, 50 µm.

**Figure 3:**
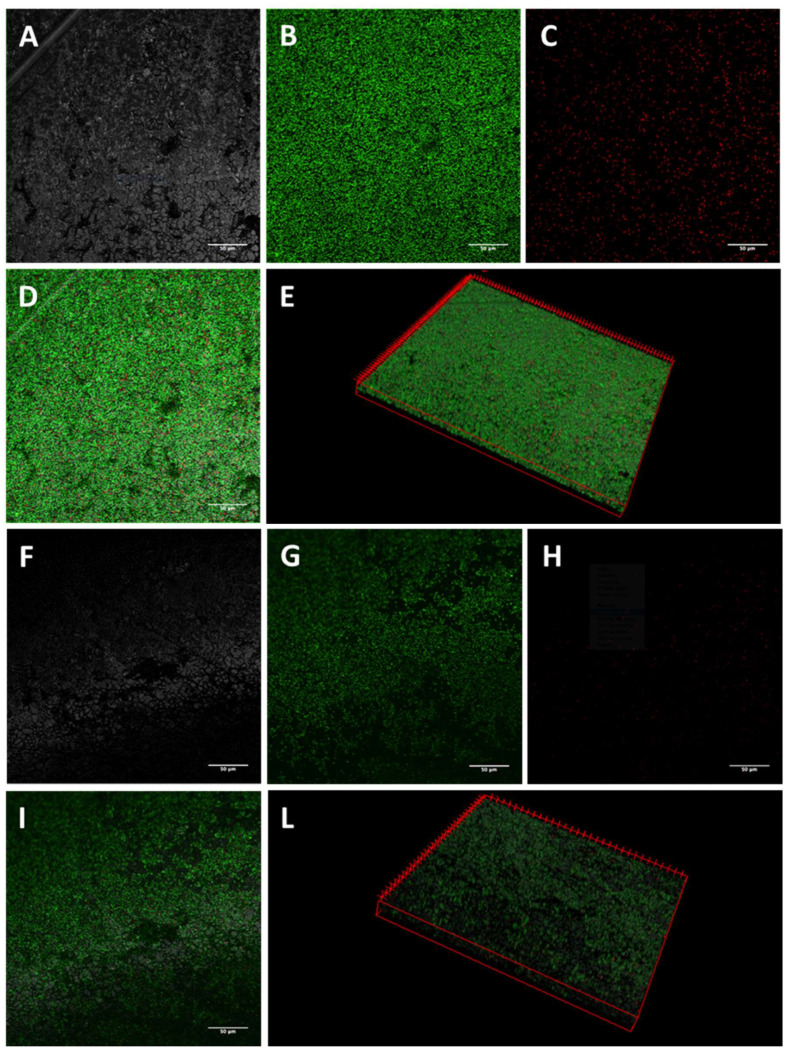
Representative images of *E. coli* FS63 biofilms obtained by CLSM. Mature biofilms formed on stainless steel surfaces were treated for 16 h with 46 µM K10T-TT (**F**–**L**) or sterile water (**A**–**E**). (**A**,**F**): stainless steel surface (reflected light); (**B**,**G**): viable cells (green fluorescence); (**C**,**H**): dead cells (red fluorescence); (**D**,**I**): merge of green and red channels; (**E**,**L**): three-dimensional reconstructions of the series along the Z-axis. Bars, 50 µm.

**Figure 4:**
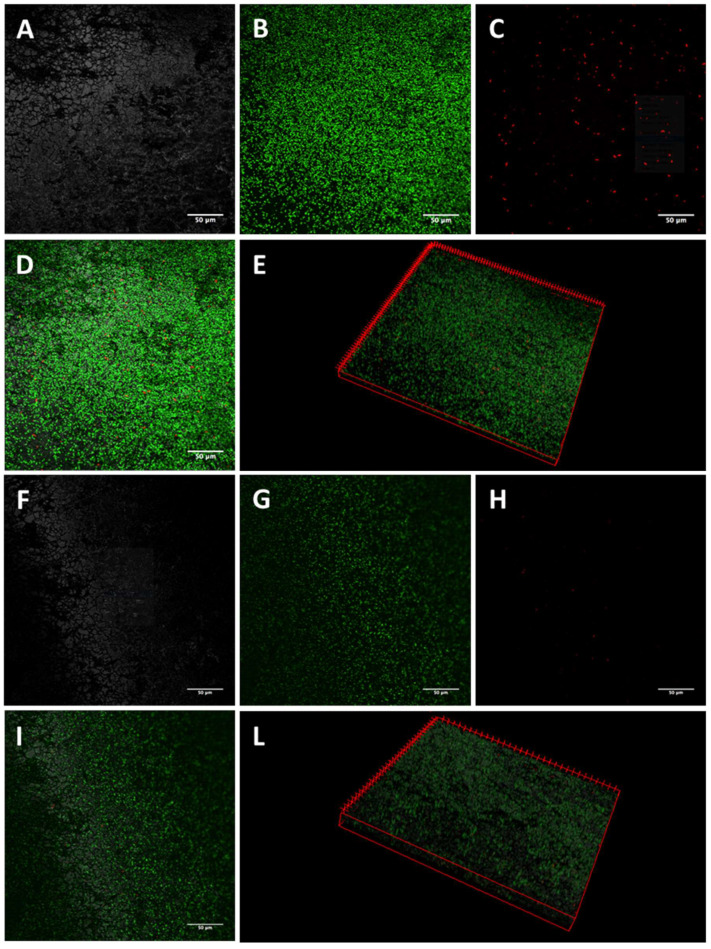
Representative images of *E. coli* CS160 biofilms obtained by CLSM. Mature biofilms formed on stainless steel surfaces were treated for 16 h with 46 µM K10T-TT (**F**–**L**) or sterile water (**A**–**E**). (**A**,**F**): stainless steel surface (reflected light); (**B**,**G**): viable cells (green fluorescence); (**C**,**H**): dead cells (red fluorescence); (**D**,**I**): merge of green and red channels; (**E**,**L**): three-dimensional reconstructions of the series along the Z-axis. Bars, 50 µm.

The activity of K10T-TT on mature biofilms formed on stainless steel by the tested *E. coli* strains was evaluated by CLSM. The images of biofilm sections and the three-dimensional (3D) reconstructions along the Z-axis of untreated *E. coli* biofilms showed mainly viable cells organized in homogeneous and tough biofilm layers (Figure 1, Figure 2, Figure 3 and Figure 4; panels A–E). The reconstructions highlighted the presence of few dead cells uniformly distributed within the biofilm and interspersed among viable cells. After K10T-TT treatment, a substantial reduction in the number of cells adhering to the stainless steel surface was observed for both the cephalosporin-sensitive *E. coli* ATCC 25922 reference strain and the ESBL-producing *E. coli* isolates (Figure 1, Figure 2, Figure 3 and Figure 4; panels F–L), demonstrating the peptide’s potential to reduce the biomass of preformed biofilms. 

**Figure 5:**
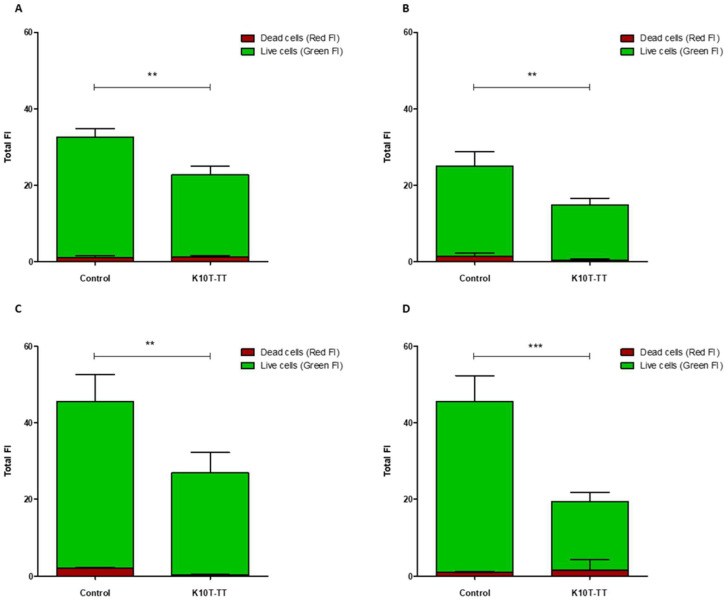
Viability of *E. coli* biofilms treated with K10T-TT. Average fluorescence intensity (FI) of live (green) and dead cells (red) quantified in three-dimensional CLSM reconstructions of *E. coli* biofilms after treatment with K10T-TT (46 µM) or water (control) for 16 h. FI measures were carried out with Imaris 9.5.0 on four random fields of each biofilm. (**A**): *E. coli* ATCC 25922; (**B**): *E. coli* FS3; (**C**): *E. coli* FS63; (**D**): *E. coli* CS160. Data are presented as mean ± standard deviation (** *p* < 0.01; *** *p* < 0.001).

A quantitative analysis of Fluoresence Intensities (FIs) of viable and dead cells, performed on the 3D CLSM reconstructions of four random fields from each sample, demonstrated that treatment with K10T-TT of preformed biofilms significantly reduced the number of viable cells in the biofilms produced by all *E. coli* strains (Figure 5). The number of dead cells showed significant variations only for *E. coli* FS63 (*p* < 0.001). Considering the total biomass (live and dead cells), following the treatment with K10T-TT average percentage biofilm reductions of 30%, 40%, 41%, and 61% were recorded for *E. coli* ATCC 25922, FS3, FS63, and CS160, respectively.

### 2.4. Activity of K10T-TT on Mature E. coli Biofilms Formed on Stainless Steel Surfaces Assessed by Scanning Electron Microscopy

**Figure 6:**
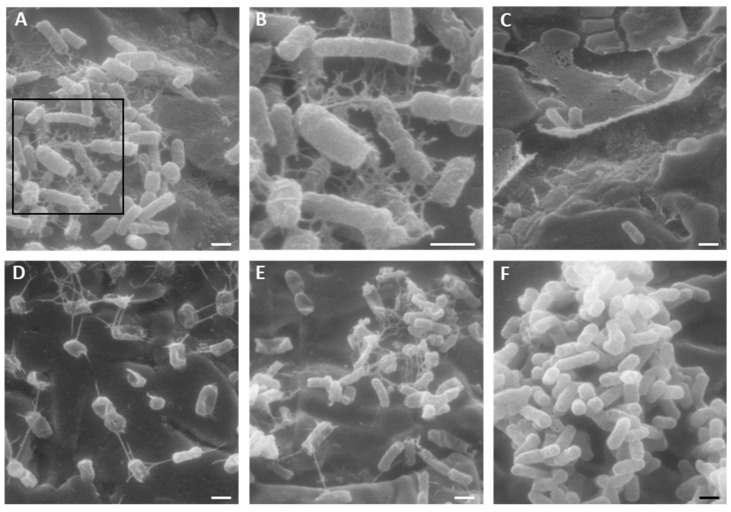
Representative scanning electron microscopy (SEM) images of mature *E. coli* biofilms on stainless steel surfaces treated for 16 h with 46 µM K10T-TT. (**A**): *E. coli* ATCC 25922; (**B**): higher magnification of the inset in panel (**A**); (**C**): *E. coli* FS3; (**D**): *E. coli* FS63; (**E**): *E. coli* CS160. (**F**): typical appearance of *E. coli* mature biofilm maintained for 16 h in sterile water (image obtained from FS3 strain). Bar = 1 µm.

The morphology of the cells of the tested *E. coli* strains in mature biofilms after treatment with K10T-TT was analyzed by SEM (Figure 6). For all the tested strains, the treatment with the peptide altered the morphology of cells. Networks due to K10T-TT aggregation on *E. coli* cells were observed, similar to those previously observed with KP [24] and K10S [25] on yeast cells.

## 3. Discussion

The spread of drug resistance among microorganisms is a steadily increasing phenomenon, posing a problem of growing importance for human health. Antimicrobial drugs are essential in treating infectious diseases, but the inappropriate and/or excessive use of these molecules in both human and veterinary medicine, as well as in animal husbandry as growth promoters, favors the selection and spread of resistant microorganisms [28]. Growing evidence of the contribution to the spread of drug resistance given by gene transfer among pathogenic and commensal microbial populations also indicates the opportunity to develop new control strategies aimed at limiting infections and colonization by resistant microorganisms in intensive farming, eliminating, or reducing environmental reservoirs [29,30,31,32]. 

The spread of resistance to critically important antibacterial drugs, such as β-lactams belonging to the class of third- and fourth-generation cephalosporins is particularly alarming [33,34]. In fact, according to the last World Health Organization report, third-generation cephalosporin-resistant *E. coli* are prioritized in the critical group of pathogens that require urgent attention [35].

Issues related to the development of new therapeutic and control approaches towards drug-resistant *E. coli* strains can be further complicated by the ability of these microorganisms to form biofilms on body surfaces and medical devices as well as environmental surfaces. In biofilms, bacteria are protected from both environmental stresses, such as dehydration or the action of disinfectants, and the action of antimicrobial drugs [36,37,38]. Moreover, it has been demonstrated that virulence genes can spread among bacteria within biofilms, as evidenced, for example, by Shiga toxin-like genes of *E. coli,* supporting the hypothesis that biofilms may also constitute an ideal environment for the evolution of new pathogens [39,40,41].

In this context, the aim of this study was to evaluate the antibacterial efficacy of selected synthetic peptides against ESBL-producing *E. coli* strains capable of forming biofilms, isolated from animal samples from intensive farming [27].

Against these ESBL-producing *E. coli* isolates, the effect of the previously described peptide KP, already known for its antimicrobial properties, and three in silico designed synthetic peptides (K10S, K10T-TT, K10S-SS) was tested. EC_50_ values obtained through CFU assays demonstrated excellent antibacterial activity for all the peptides against planktonic cells of both a reference strain of *E. coli* sensitive to cephalosporins and the ESBL-producing isolates (Table 2). 

A fundamental characteristic of antimicrobial compounds potentially useful in human and veterinary medicine is the absence of toxicity towards higher eukaryotic cells. This characteristic has been widely demonstrated for KP in vitro against various cell lines and in vivo in murine models [24]. The absence of hemolytic and cytotoxic effects was also demonstrated for the other investigated peptides [25,26], suggesting their potential for the development of new anti-infective drugs. 

Among the studied peptides, K10T-TT and K10S-SS were able to inhibit the initial stages of biofilm formation by the reference strain and the ESBL-producing isolates of *E. coli* (Table 3), as demonstrated using a widely diffuse 96-well microtiter plate assay based on the crystal violet dye that marks polysaccharides of the extracellular matrix and peptidoglycan of live and dead bacterial cells [42]. By contrast, the peptides KP and K10S, derived from a single amino acid substitution in the KP sequence, demonstrated poor activity against bacterial biofilms. This different behavior may be attributed to the greater number of amino acid substitutions in K10T-TT and K10S-SS sequences, including charged residues along the peptide chain, that could result in different interactions of the peptide with the polysaccharide matrix of the biofilm [25,26]. For K10T-TT and K10S-SS, peptide concentrations showing activity against biofilms were about 100 times higher than those effective against planktonic *E. coli*. This observation confirms the protective effect of biofilm against bacterial cells, in line with previous studies showing reductions in the activity of various antimicrobials by 10 to 1000 times against biofilms compared to planktonic cells [43,44,45]. In any event, the inhibitory effect on biofilm formation obtained in this study is significant and suggests the possible use of these peptides in formulations to directly coat or treat different surfaces, including medical devices such as urinary and central catheters, for preventing colonization by biofilm-producing microorganisms.

To verify the possibility of using peptides also for the eradication of preformed biofilms, the most promising peptide, K10T-TT, was further studied using a CLSM approach to evaluate its bactericidal and detachment activity within the context of a mature biofilm developed on stainless steel surfaces, used as a model of orthopedic implants and prosthetic joints, as well as for environmental contamination in slaughterhouses. The analysis of 3D reconstructions of CLSM images of biofilms by quantitative measures of fluorescence intensities has been demonstrated as reliable as CFU counts through validation experiments across a range of bacterial species [46,47]. In this study, the results revealed, after treatment with the peptide, a reduction in the number of viable cells within biofilms formed by both the reference strain and the ESBL-producing *E. coli* isolates. In most cases, the number of dead cells did not undergo significant variations, while they decreased for one strain. This is probably due to the detachment of non-viable cells following treatment (Figure 5). 

SEM studies showed an alteration in cellular morphology in isolates treated with the K10T-TT peptide (Figure 6). Furthermore, the images highlighted the presence of networks due to peptide aggregation on *E. coli* cells, similarly to what was previously observed on *Candida albicans* cells treated with KP [24] and K10S peptides [25]. It has been demonstrated that KP can dimerize under nonreducing conditions due to the formation of intermolecular disulfide bridges between cysteine residues at position 7 capable of stabilizing its structure. Subsequently, through circular dichroism studies, it was verified that KP dimers can spontaneously and reversibly interact and aggregate to form an organized network of fibrillar structures, and that this process is catalyzed by soluble or yeast cell surface-exposed β-1,3-glucans [24]. Many of the biological activities of KP appear to be associated with these chemical-physical and structural characteristics. Self-aggregation could protect the peptide in vivo from protease action, and slow dissociation kinetics could ensure the release of the active dimeric form over time [24]. While the K10S peptide was not able to spontaneously undergo any transition toward a recognizable organized structure [25], all its derivatives were able to acquire a well-defined secondary structure. In particular, a β-sheet structure was observed for K10T-TT, while K10S-SS showed an α-helix conformation [26].

The largest group of AMPs with a known secondary structure are α-helical peptides, which are linear in solution and form amphipathic helices upon interaction with bacterial membranes, leading to pore formation and rapid cell lysis at high concentrations [48,49,50]. At lower concentrations, some α-helical peptides can prevent biofilm formation through mechanisms like quorum-sensing inhibition. For example, the human cathelicidin LL-37 has been shown to down-regulate the transcription of two major quorum-sensing systems, Las and Rhl [51,52]. 

In contrast, β-sheet peptides like K10T-TT are stabilized by disulfide bridges and do not undergo major conformational changes upon interaction with membranes [48,49]. They typically disrupt membranes through a carpet-like mechanism, where they cover the membrane surface similarly to a carpet and destroy the cell membrane in a detergent-like manner [23]. This mechanism may also destroy the membranes of bacterial cells within biofilms. However, more specific mechanisms may also be at play, as seen with an engineered antibacterial β-sheet peptide effective against multidrug-resistant *Salmonella* Typhimurium. This peptide likely exerts antibiofilm activities through the formation of complexes with bacterial DNA, inhibition of fimbriae and flagella synthesis and motility, and interference with autoinducer-2 (AI-2)-mediated quorum sensing, eventually leading to reduced viable cells within biofilms [53].

Although the target of K10T-TT on *E. coli* cells is yet to be verified, the presence of a cysteine in the sequence and the formation of fibrillar structures in the presence of bacteria suggest that this peptide shares significant analogies with KP, which may be important for its biological activity and potential applications.

The results obtained, albeit preliminary, could indicate that the peptides under study are possible candidates for the development of new effective antimicrobials against drug-resistant *E. coli* strains. Moreover, K10T-TT may represent a leading candidate for further research aimed at investigating its antibiofilm mechanism of action, including interactions with target cells or molecules and the specific cellular pathways or processes it affects.

## 4. Conclusions

The escalating issue of antimicrobial resistance, particularly in ESBL-producing *E. coli*, underscores the urgent need for new therapeutic strategies. In this study, antifungal peptides derived from KP were shown for the first time to display a significant antibacterial and antibiofilm activity against cephalosporin-resistant *E. coli* strains, with K10T-TT showing the most promise. Although further research is necessary to fully elucidate its mechanisms of action, this peptide holds potential for the development of effective alternatives in combating resistant *E. coli* infections and biofilm-related complications.

## 5. Materials and Methods

### 5.1. Bacterial Strains

A reference *E. coli* ATCC 25922 strain and four ESBL-producing *E. coli* strains isolated from feces and carcasses of pigs bred in intensive farms (FS3, FS63, and CS160) were used in this study [27]. Bacterial strains were maintained on Mueller–Hinton Agar (MHA; Sigma-Aldrich, St. Louis, MO, USA) plates. Prior to each experiment, fresh cultures were prepared on MHA plates incubated at 37 °C overnight. 

### 5.2. Antimicrobial Peptides

The investigated peptides were synthesized by the solid phase method at the CRIBI Biotechnology Center (University of Padua, Padua, Italy). The peptides were solubilized in dimethyl sulfoxide (DMSO) at a concentration of 20 mg/mL, stored at 4 °C, and diluted in sterile distilled water at experimental concentrations. Controls were prepared without peptides and always contained DMSO at proper concentrations.

### 5.3. Bactericidal Activity against Planktonic Cells

The antibacterial activity of the peptides against *E. coli* ATCC 25922 and EsβL-producing *E. coli* isolates was evaluated by CFU assay, as previously described, with minor modifications [54].

Briefly, bacterial cells were cultured on MHA plates at 37° C for 24 h, then diluted to 2 × 10^3^ cells/mL in 100 μL of sterile distilled water with the peptide at serial dilutions (20–0.2 μM). Cells diluted in water served as controls. After 5 h at 37 °C, bacterial suspensions were plated on MHA, and colonies were counted after 24 h of further incubation at 37 °C. Percent killing was determined relative to the number of colonies in controls. Each assay was conducted in triplicate, and at least two independent experiments were carried out for each condition. EC_50_ values were calculated using Prism 5 software (Graph Pad, San Diego, CA, USA) through nonlinear regression analysis.

### 5.4. Inhibition of Biofilm Formation on Polystyrene Surfaces

The effects of the peptides on early stages of biofilm formation by all the tested *E. coli* strains on polystyrene plates were investigated as previously described, with minor modifications [55]. Bacterial cells were grown in Tryptic Soy Broth (TSB, Sigma-Aldrich) at 37 °C, 150 rpm, for 18 h. Broth cultures were diluted to 5 × 10^6^ cells/mL in fresh TSB and transferred (200 μL/well) into flat-bottom 96-well plates (Corning Incorporated, New York, NY, USA). After incubation for 90 min at 37 °C, planktonic bacteria were removed by washing with phosphate-buffered saline (PBS). Adherent cells were then exposed to serial dilutions of peptides (100–10 μM) in 200 µL of sterile distilled water for 5 h at 37 °C, while cells incubated in water served as the control. Following treatment, wells were washed and replenished with 200 μL of fresh TSB, and the plates were further incubated at 37 °C for 24 h. Each assay was performed in triplicate, and the process was repeated in three independent experiments. The biomass of treated and control biofilms was assessed using the CV assay (Sigma-Aldrich, St. Louis, MO, USA). The plates were washed three times with PBS and then dried at 80 °C for 15 min before staining with 200 µL/well of 0.25% CV for 15 min. After washing and drying, 200 µL/well of 85% ethanol was added, and the absorbance at 540 nm was measured after 15 min using a microplate reader (Multiskan Ascent Microplate Reader, Thermo Electron, Waltham, MA, USA). The results were expressed as a percentage of the reduction in biofilm mass relative to untreated controls. The respective EC_50_ values were calculated using Graph Pad Prism 5 software, as described previously.

### 5.5. Inhibition of Mature E. coli Biofilms on Stainless Steel Surfaces

The activity of the most promising peptide, K10T-TT, on preformed biofilms was evaluated using an in vitro model of contamination of stainless steel surfaces. Sterilized steel squares (1 cm^2^, 0.1 cm thickness) placed in the wells of flat-bottom 24-well plates were inoculated with 500 μL of a 5 × 10^6^ cells/mL suspension of *E. coli* (ATCC 25922 or EsβL-producing strains) prepared by dilution of overnight broth cultures in fresh TSB. After 24 h of incubation at 37 °C, the medium was gently washed off and the samples were treated with 500 μL/well of K10T-TT (46 µM) or sterile water (controls) for 16 h at 37 °C. The concentration of K10T-TT used in these experiments was chosen as the average EC_50_ observed in the CV assay, excluding the outliers. The effect of K10T-TT on the biofilms preformed on stainless steel surfaces was assessed by CLSM and SEM. Results were obtained from two independent experiments.

#### 5.5.1. Confocal Laser Scanning Microscopy

Biofilms formed on stainless steel surfaces and treated with K10T-TT were analyzed using CLSM to assess biofilm architecture and bacterial viability in different biofilm layers. After washing with PBS, the samples were stained with 500 μL of a live/dead kit (LIVE/DEAD FilmTracer™ Biofilm Viability Kit, Invitrogen, Paisley, UK) solution containing 0.3% SYTO-9 and 0.3% propidium iodide (PI) following the manufacturer’s instructions. After 20 min, the biofilms were washed again, and fluorescence emission was detected using an LSM 510 Meta scan head integrated with an Axiovert 200 M inverted microscope (Carl Zeiss, Jena, Germany). The excitation/emission wavelengths were 480/500 nm for the SYTO-9 live cell stain and 490/635 nm for the PI dead cell stain. Samples were observed using a 40× NA1.3 oil immersion lens, and four random fields were scanned in each sample. Three independent experiments were performed. A stack of 80–100 slices at 0.5 μm step sizes was captured along the Z-axis of the biofilm. CLSM images were acquired, and 3D reconstructions were generated using the Axiovision software module Inside 4D release 4.5 (Carl Zeiss, Jena, Germany). The ratio of red fluorescence intensity (FI) to combined green-and-red FI, calculated with Imaris 9.5.0 software (Bitplane AG, Zurich, Switzerland), was used to determine the proportion of dead cells in the treatment groups. Statistical analysis was conducted using Prism 5 (Graph Pad software, San Diego, MA, USA). A Student’s *t*-test was used to compare the means of two groups. Values of *p* < 0.05 were considered significant.

#### 5.5.2. Scanning Electron Microscopy

SEM analysis was carried out to evaluate the effect of K10T-TT on mature biofilms on stainless steel. After treatment, the samples were washed with PBS, dried for 15 min at room temperature, and fixed with a solution of 2.5% glutaraldehyde in 0.1 M sodium cacodylate for 1 h at room temperature. The samples were then dehydrated through a graded series of ethanol (25%, 50%, 75%, 90%, and 100%, 30 min for each concentration), immersed in absolute acetone, and subjected to critical point drying. The samples were mounted on aluminum stubs and coated with a 60 nm gold film using a metal sputtering device. Observation was performed using a Philips 501 microscope equipped with a Nikon Coolpix digital camera for image acquisition.

## Figures and Tables

**Table 1:** Amino acid sequences and characteristics of the investigated peptides.

Peptide	Sequence	MM (Da)	pI	Charge	AI	GRAVY	Ref.
KP (A10S)	AKVTMTCSAS	998.18	8.27	1+	49	0.53	[24]
K10S	**K**KVTMTCSAS	1055.27	9.31	2+	39	−0.04	[25]
K10T-TT	KKVTMTC**T**A**T**	1083.33	9.31	2+	39	−0.02	[26]
K10S-SS	KKV**S**M**S**CSAS	1027.22	9.31	2+	39	−0.06	[26]

MM (Da), molecular mass (Dalton); pI, isoelectric point; AI, aliphatic index; GRAVY, grand average of hydropathy. In bold, substituted residue; MM, pI, charge, AI and GRAVY calculated by ExPASy tool ProtParam.

**Table 2:** In vitro bactericidal activity of the investigated peptides against planktonic *E. coli* strains.

*E. coli* Strain	EC_50_^1^ (95% Confidence Intervals)			
	KP	K10S	K10T-TT	K10S-SS
ATCC 25922	0.309 (0.233–0.375)	0.347 (0.282–0.427)	0.394 (0.312–0.497)	0.195 (0.183–0.209)
FS3	0.848 (0.827–0.870)	0.139 (0.133–0.146)	0.385 (0.367–0.405)	0.587 (0.540–0.639)
FS63	0.751 (0.732–0.770)	0.051 (0.033–0.079)	0.475 (0.427–0.529)	0.203 (0.201–0.205)
CS160	1.099 (1.059–1.142)	0.998 (0.724–1.377)	0.607 (0.488–0.756)	0.149 (0.137–0.162)

^1^ EC_50_, half-maximal effective concentration (M × 10^−6^).

**Table 3:** In vitro activity of the investigated peptides against biofilm formation by different *E. coli* strains on polystyrene plates.

*E. coli* Strain	EC_50_^1^ (95% Confidence Intervals)	
	K10T-TT	K10S-SS
ATCC 25922	49.569 (31.491–78.039)	89.442 (39.212–204.00)
FS3	13.792 (4.863–39.093)	26.187 (5.615–122.17)
FS63	44.109 (19.288–100.87)	55.569 (9.346–330.45)
CS160	56.42 (54.566–58.335)	69.062 (51.205–93.135)

^1^ EC_50_, half-maximal effective concentration (M × 10^−6^).

## Data Availability

The raw data supporting the conclusions of this article will be made available by the authors on request.
